# ﻿*Melanoseriskangdingensis* (Lactucinae, Cichorieae, Asteraceae), a new species reported from western Sichuan, China

**DOI:** 10.3897/phytokeys.236.113401

**Published:** 2023-11-24

**Authors:** Qian-Qian Zhong, Ze-Huan Wang, Jia-Ju Xu, Qin-Wen Sun

**Affiliations:** 1 Department of Traditional Chinese Medicine Resources and Development, College of Pharmacy, Guizhou University of Traditional Chinese Medicine, Guiyang 550025, Guizhou, China Guizhou University of Traditional Chinese Medicine Guiyang China

**Keywords:** *Melanoserisbracteata*, *Melanoserismacrantha*, morphology, new taxon, taxonomy

## Abstract

*Melanoseriskangdingensis*, a new species native to western Sichuan, China, is firstly described and illustrated, and its conservation status is also assessed. It bears resemblance to *M.macrantha* and *M.bracteata* in terms of morphology; however, there are distinguishing characteristics in terms of their leaf structure, presence of bracts, hairiness of involucre, number of florets, and length of both stamen tube and achene’s beak.

## ﻿Introduction

*Melanoseris* Decne. is a genus belonging to the subtribe Lactucinae, tribe Cichorieae of the Asteraceae family. This genus was first established by Decaisne (1843) based on two species, namely *M.lessertiana* (Wall. ex DC.) Decne. and *M.lyrata* Decne., but it has now become the largest genus of the subtribe Lactucinae in China. After extensive research conducted by plant taxonomists (Decaisne 1843; Edgeworth 1846; Shih 1991, 1997; Zhu 2004; Zhu et al. 2004, 2006; Bano 2009; Bano and Qaiser 2009, 2010, 2011; Kilian et al. 2009, 2017; Wang et al. 2009; Deng et al. 2011; Shih and Kilian 2011; Zhang et al. 2011; Wang et al. 2013, 2015, 2020; Abid et al. 2017; Yin et al. 2018), there has been a significant improvement in our understanding of the morphological characteristics of *Melanoseris* species and their relationships within the genus. According to the most recent delimitation of the genus and species (Wang et al. 2013, 2015, 2020; Yin et al. 2018), there are currently 19 accepted species of *Melanoseris* known to occur in China.

During a thorough examination of all the *Melanoseris* specimens in the herbaria, the corresponding author discovered scans of two intriguing specimen images (Gao Yundong et al. THP–KD–2024 at CDBI). These particular specimens had previously been identified as *M.macrantha* (C.B.Clarke) N.Kilian & J.W.Zhang, but what caught their attention was the presence of densely covered long white trichomes on the involucre, setting it apart from all other known *Melanoseris* species. To investigate the stability of this unique characteristic, a field investigation was conducted based on the collection information obtained from the specimens. Two populations of this species were discovered, and all the observed plants in the flowering and fruiting stages exhibited densely covered involucres with these long white trichomes. This confirmed that the indumentum of the involucre is indeed a stable and distinct feature. Further morphological studies and analysis revealed similarities between this plant and *M.macrantha* and *M.bracteata* (Hook.f. & Thomson ex C.B.Clarke) N.Kilian, but also distinct differences. Therefore, the authors concluded that this plant represents a new species, which is described and illustrated in detail in this study.

## ﻿Materials and methods

The morphological description of the new species was conducted based on live plants that were observed and photographed in the field. Additionally, herbarium collections (KUN, GTZM) from these occasions were utilized. To compare the morphology, we referred to the keys and descriptions for the genus and species in Flora Reipublicae Popularis Sinicae (Shih 1997) and Flora of China (Shih and Kilian 2011). To facilitate further comparison, we examined specimens and photographs in the herbaria of Chengdu (CDBI), Kunming (KUN), and Beijing (PE). The morphology of trichomes and pappus, as well as the length of ligules, anther tubes, and achenes, were observed or measured using anatomy microscope (OD500H) or light microscope (Olympus DP72) on fresh or pickled flowers or achenes. Photographs were taken using a Canon EOS 77D and a Dell E2014Hf camera. Figures were edited, arranged, and merged using Adobe Illustrator CS4. The conservation status is determined based on the actual population size observed in the field and the assessment criteria of the IUCN.

## ﻿Results

### ﻿Taxonomy

#### ﻿ Melanoseris kangdingensis

Taxon classificationPlantaeAsteralesAsteraceae

Ze H.Wang sp. nov.

B2BEE304-48A2-5196-AFBA-0239247CB589

urn:lsid:ipni.org:names:77331502-1

Fig. 1

##### Type.

China, Sichuan Province, Ganzi Tibetan Autonomous Prefecture, Kangding City, Pusharong Town, Kuxirong Village. 29°25.63'N, 101°18.39'E, alt. 2848 m, 22 Aug 2023, Wang Zehuan, Zhong Qianqian & Xu Jiaju wzh20230801 (holotype: KUN!, isotypes: KUN!, LBG!).

##### Diagnosis.

*Melanoseriskangdingensis* most closely resembles *M.bracteata* in the presence of subequal phyllaries, and numerous peduncle bracts grading into the narrow outer phyllaries, but differs from the latter in basal leaves persistent (vs. wither) during flowering, leaves noticeably pinnatipartite (vs. typically entire), phyllaries densely covered with multiseriate glandular hairs (vs. glabrous), length of anther tubes 5.5–6.4 mm (vs. 3.7–4.6 mm), achenes 10–11 mm (vs. 7.9–8.5 mm), beak ca. 4 mm, about 1/2 length of the achene’s body (vs. ca. 6.5 mm, nearly equal in length to the achene’s body).

**Figure 1.**
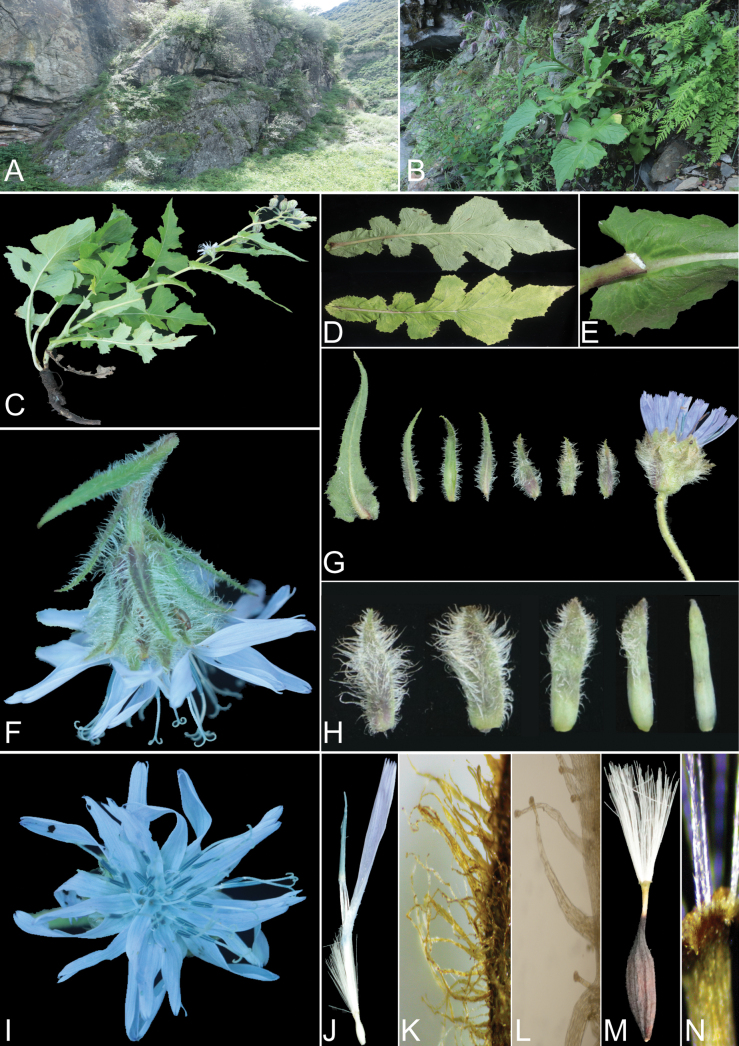
*Melanoseriskangdingensis***A, B** habitat **C** plant **D** surface of leaves **E** petiole base **F** lateral view of the capitulum **G** bracts on peduncular branches and one capitulum **H** phyllaries of each layer **I** upper view of the capitulum **J** ray floret **K** trichomes on the outer phyllary **L** microscope photos of trichomes, to show its type (multiseriate glandular hairs) **M** achene **N** the apex of beak. Photographed by Zehuan Wang.

##### Description.

Perennial herb, 30–80 cm tall. Roots about 1–2 cm in diameter, fleshy, cylindrical, often branched. Stems erect, rather robust. Basal leaves 21–45 × 10–19 cm, persist in flowering, elongated oblong, lyrately pinnatipartite; terminal lobes 5.5–22 × 5–19 cm, broadly triangular or broadly ovate, apex acuminate; lateral lobes 2–3 pairs, 5.2–10.3 × 3.4–6 cm, triangular, ovate or rectangular, apex obtuse or truncate. Leaf margins coarsely dentate, green on both sides, sparsely covered with multiseriate glandular hairs; petiole 5–10 cm long, sparsely covered with multiseriate glandular hairs. Middle and lower stem leaves 8–28 × 3.5–12 cm, homomorphic with basal leaves, pinnatipartite; terminal lobes 3–9 × 3.5–12 cm, elongated triangular, apex acuminate; lateral lobes 2–4 pairs, 2–6.5 × 1.7–5.2 cm, triangular, semicircular or rectangular, apex obtuse or truncate, leaf margins coarsely dentate, green on both sides, sparsely covered with multiseriate glandular hairs; petiole base auriculately clasping, with wings 0.5–3 cm wide. Stem leaves gradually decrease upward, transitioning into bract-like structures on the branches of the capitulescence. Lower leaves on the branches of capitulescence 7–17 × 2.6–7.5 cm, elongated oblong or lanceolate, deeply or shallowly pinnatilobed, or entire, apex long acuminate, leaf margins coarsely dentate, sparsely hairy. Bracts on a capitulescence branches 4–7, 1.5–5.5 × 0.1–1.5 cm, lanceolate or linear, gradually receding to involucral bracts, margin entire, green on both sides, dorsal and margin densely covered with white flattened multiseriate glandular hairs. Capitulescence corymbiform, branches slender, peduncle 2–9.5 cm long, densely covered with long multiseriate glandular hairs. Capitula 4–10, pendulous in flowering, with 22–27 florets. Involucre 1.9–2.2 × 1.3–1.8 cm, broadly campanulate, densely covered with long white multiseriate glandular hairs abaxially and along the margin. Phyllaries 5–seriate, subequal, apical acute or obtuse, margin entire. Outer phyllaries ca. 16 × 2 mm, oblong, slightly shorter than inner phyllaries, purple or purplish-green, densely covered with broadly flat multiseriate glandular hairs abaxially or along the margin, trichomes up to ca. 2 mm long; middle phyllaries ca. 16 × 1.5 mm, broadly linear, green or apically purplish, with multiseriate glandular hairs gradually decreasing from the outer to inner layers; innermost phyllaries linear, ca. 16 × 1 mm, light green, subapical sparsely with multiseriate glandular hairs. Florets ligulate, tube ca. 9 mm long, white; ligules ca. 17 × 2 mm, 5–toothed at the apex, light blue. Stamens synantherous, anther tube 5.5–6.4 mm long, light blue. Ovary inferior, flattened, ellipsoid, style ca. 22 mm long, apically bifid, stigmatic branches ca. 1.5 mm long, long and acuminate, evenly coated with elongate collecting hairs. Achenes 10–11 × 1.5–2 mm, fusiform, dark brown, compressed, lateral ribs slightly thickened, each side with 3 slightly raised ribs, surface sparsely hairy, and apex contracted into ca 4 mm beak, beak discolorous, with the top half being white. Pappus ca. 8 mm long, white, consisting of a single layer, finely serrated.

##### Distribution and habitat.

*Melanoseriskangdingensis* is currently known from two localities in western Sichuan, China. It has been observed growing on the slope and at the foot of cliffs by the roadside at elevations ranging from 2800–2900 m. The dominant species of the community include *Dasiphorafruticosa* (L.) Rydb. (Rosaceae), *Gentianatibetica* King ex Hook.f. (Gentianaceae), *Heracleumcandicans* Wall. ex DC. (Apiaceae), *Paraceterachvestita* (Hooker) R.M.Tryon (Pteridaceae), and *Cheilantheschusana* Hook. (Pteridaceae).

##### Phenology.

Flowering and fruiting from July to September.

##### Etymology.

The specific epithet ‘*kangdingensis*’ refers to Kangding City in the Sichuan Province, which is the locality of the type collection.

##### Vernacular name.

Simplified Chinese: 康定毛鳞菊; Chinese Pinyin: Kāngdìng Máolínjú.

##### Conservation status.

Currently, two populations of *Melanoseriskangdingensis* have been discovered, each with approximately 20 mature individuals. These plants grow on slopes and at the foot of cliffs by the roadside, making them vulnerable to disturbance from human activities. Additionally, the habitat and dispersal environment for reproduction are steep and harsh, making it challenging for the species to expand its distribution area. Based on the IUCN Red List criteria (IUCN 2012, 2022), this new species should be classified as Endangered (EN; criteria B1ac(iii)+2ac(iii); C2a(i); D). However, we recognize that further assessments are required as additional populations are identified.

##### Additional specimens examined.

China, Sichuan Province, Ganzi Tibetan Autonomous Prefecture, Kangding City, Pusharong Town, Binggu Village, 29°28.18'N, 101°18.84'E, alt. 2939 m, 22 Aug 2023, Wang Zehuan, Zhong Qianqian & Xu Jiaju wzh20230802 (KUN!, GTZM!); China, Sichuan Province, Ganzi Tibetan Autonomous Prefecture, Kangding City, Pusharong Town, Kuxirong Village, 29°25.07'N, 101°18.02'E, alt. 2800–3000 m, 04 Aug 2017, Gao Yundong, Deng Hengning & Li Huaicheng THP–KD–2024 (CDBI!).

## ﻿Discussion

**Figure 2.**
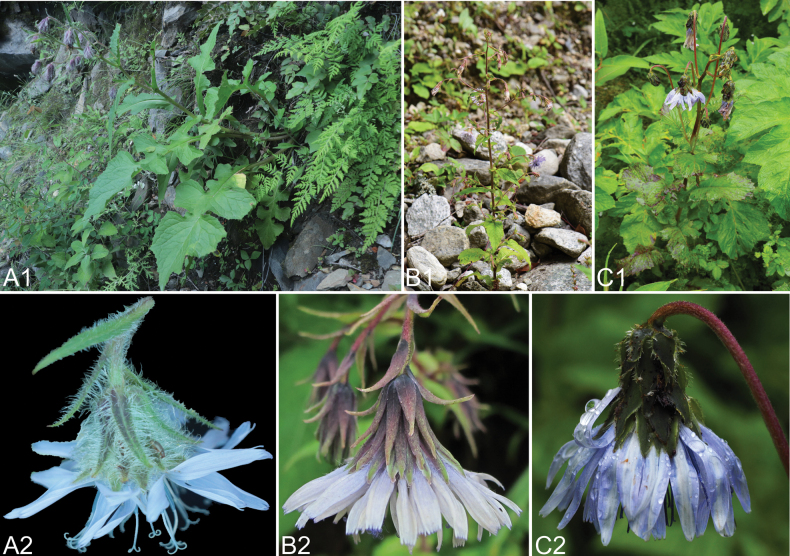
Photographs of the plants and capitula of *Melanoseriskangdingensis*, *M.bracteata* and *M.macrantha***A1, A2***M.kangdingensis***B1, B2***M.bracteata***C1, C2***M.macrantha*. Photographed by Zehuan Wang.

The main distinguishing feature between *Melanoseriskangdingensis* and *M.bracteata* is the dense coverage of flat, long multiseriate glandular hairs on the back and margins of the outer phyllaries (Fig. 2). Another species, *M.macrantha*, also possesses similar flat trichomes on the outer phyllaries. However, *M.kangdingensis* can be easily differentiated by its numerous and conspicuous bracts gradually transitioning into the outer phyllaries, fewer florets in the capitulum, subequal phyllaries densely covered with multiseriate glandular hairs abaxially, longer anther tube length, and beak. A summary of the main morphological differences between *M.kangdingensis*, *M.macrantha*, and *M.bracteata* is provided in Table 1. In fact, *M.kangdingensis* is the species with the hairiest involucre among the *Melanoseris* genus. Fluffy and hairy involucre may be related to the specific growing environment of *M.kangdingensis*. Most *M.kangdingensis* plants grow on wind-exposed cliffs without tall vegetation, and the dense hairy involucre serves to better protect the smooth development of the achenes inside.

**Table 1.** Morphological comparison between *M.kangdingensis*, *M.macrantha*, and *M.bracteata*.

Characters	*M.kangdingensis*	*M.macrantha*	*M.bracteata*
**Leaves**	basal leaves persistent during flowering; leaves pinnatipartite	basal leaves wither during flowering; leaves pinnatipartite	basal leaves wither during flowering; leaves usually entire
**Bracts**	numerous and conspicuous, grading into the narrow outer phyllaries	absent	numerous and conspicuous, grading into the narrow outer phyllaries
**Capitula**	with 22–27 florets	with ca. 40 florets	with usually 20–30 florets
**Involucres**	phyllaries subequal, narrow, densely covered with long white multiseriate glandular hairs abaxially and along the margins, trichomes gradually decreased from the outer to inner phyllaries	phyllaries imbricate, outer phyllaries wide, margin white densely fimbriate, glabrous abaxially; inner phyllaries narrow, completely glabrous	phyllaries subequal, narrow, all completely glabrous
**Anther tube length**	5.5–6.4 mm	3.7–4.3 mm	3.7–4.6 mm
**Achene length**	10–11 mm	10–11 mm	7.9–8.5 mm
**Achene beak**	ca. 4 mm long, about 1/2 length of the achene’s body	ca. 1.8 mm long, about 1/3 length of the achene’s body	ca. 6.5 mm long, nearly equal in length to the achene’s body

During field surveys, it was observed that in the type population, which is situated on relatively shady slopes, only 1–2 mature achenes developed in each capitulum of several fruiting plants, while the majority of achenes or a few entire capitula were found to be sterile. Furthermore, we also discovered insect eggs inside some nearly matured achenes of the capitula, indicating that *Melanoseriskangdingensis*, like *Sinoserismuliensis* (Y.S.Chen, L.S.Xu & R.Ke) Ze H.Wang, N.Kilian & H.Peng (Wang et al. 2020), is susceptible to seed parasitism by certain insects. *M.kangdingensis* exhibits the longest anther tube length (5.5–6.4 mm) among the *Melanoseris* genus. The longer anther tubes have the potential to release a greater quantity of pollen, theoretically. A higher quantity of pollen can increase the probability of successful fertilization of the ovary, enhance seed maturation, and ultimately improve seed reproduction success. However, due to limited distribution information, further investigation into its growth and reproductive mechanisms is necessary to gain a better understanding of the adaptability and conservation requirements of this plant.

## Supplementary Material

XML Treatment for Melanoseris kangdingensis

## ﻿Additional information

### Author ORCIDs

Qian-Qian Zhong https://orcid.org/0009-0002-7051-1503

Ze-Huan Wang https://orcid.org/0000-0002-7369-5005

Jia-Ju Xu https://orcid.org/0009-0004-2852-0611

Qin-Wen Sun https://orcid.org/0000-0003-0848-5017

### Data availability

All of the data that support the findings of this study are available in the main text.
